# Intra-tumor L-methionine level highly correlates with tumor size in both pancreatic cancer and melanoma patient-derived orthotopic xenograft (PDOX) nude-mouse models

**DOI:** 10.18632/oncotarget.24264

**Published:** 2018-01-17

**Authors:** Kei Kawaguchi, Qinghong Han, Shukuan Li, Yuying Tan, Kentaro Igarashi, Kentaro Miyake, Tasuku Kiyuna, Masuyo Miyake, Bartosz Chemielwski, Scott D. Nelson, Tara A. Russell, Sarah M. Dry, Yunfeng Li, Arun S. Singh, Mark A. Eckardt, Michiaki Unno, Fritz C. Eilber, Robert M. Hoffman

**Affiliations:** ^1^ AntiCancer, Inc., San Diego, CA, USA; ^2^ Department of Surgery, University of California, San Diego, CA, USA; ^3^ Department of Surgery, Graduate School of Medicine, Tohoku University, Sendai, Japan; ^4^ Division of Hematology-Oncology, University of California, Los Angeles, CA, USA; ^5^ Department of Pathology, University of California, Los Angeles, CA, USA; ^6^ Division of Surgical Oncology, University of California, Los Angeles, CA, USA; ^7^ Department of Surgery, Yale School of Medicine, New Haven, CT, USA

**Keywords:** recombinant methionine (rMETase), methionine dependence, tumor methionine, pancreatic cancer, melanoma

## Abstract

An excessive requirement for methionine (MET) for growth, termed MET dependence, appears to be a general metabolic defect in cancer. We have previously shown that cancer-cell growth can be selectively arrested by MET restriction such as with recombinant methioninase (rMETase). In the present study, we utilized patient-derived orthotopic xenograft (PDOX) nude mouse models with pancreatic cancer or melanoma to determine the relationship between intra-tumor MET level and tumor size. After the tumors grew to 100 mm^3^, the PDOX nude mice were divided into two groups: untreated control and treated with rMETase (100 units, i.p., 14 consecutive days). On day 14 from initiation of treatment, intra-tumor MET levels were measured and found to highly correlate with tumor volume, both in the pancreatic cancer PDOX (*p*<0.0001, R^2^=0.89016) and melanoma PDOX (*p*<0.0001, R^2^=0.88114). Tumors with low concentration of MET were smaller. The present results demonstrates that patient tumors are highly dependent on MET for growth and that rMETase effectively lowers tumor MET.

## INTRODUCTION

Cancer cells have an elevated requirement for methionine (MET) compared to normal cells. This phenomena is termed MET dependence [1]. MET restriction arrests tumor growth and induces a selective S/G_2_-phase cell-cycle arrest of cancer cells *in vitro* and *in vivo* [2–5].

MET dependence appears to be due to excess use of MET for aberrant transmethylation reactions, termed the Hoffman effect [6–11], analogous to the Warburg effect for glucose in cancer [12]. The excessive and aberrant use of MET in cancer is observed in [^11^C] MET PET imaging, where high uptake of [^11^C] MET results in a very strong and selective tumor signal compared with normal tissue background. [^11^C] MET is superior to [^18^C] fluorodeoxyglucose (FDG)- PET for PET imaging, suggesting MET dependence is more tumor-specific than glucose dependence [13–15].

A purified MET cleaving enzyme, methioninase (METase), from *Pseudomonas putida* has been found previously to be an effective antitumor agent *in vitro* as well as *in vivo* [16–19]. For the large-scale production of METase, the gene from *P. putida* has been cloned in *Escherichia coli* and a purification protocol for recombinant METase (rMETase) has been established with high purity and low endotoxin [20–25].

We previously reported on the efficacy of rMETase against a BRAF-V600E mutant melanoma patient-derived orthotopic xenograft (PDOX) nude mouse model and that rMETase sensitized the melanoma PDOX to temozolomide (TEM) [26].

In the present study, we used PDOX nude mouse models with pancreatic cancer and melanoma to demonstrate the relationship between intra-tumor MET level and tumor size, using rMETase to lower tumor MET.

## RESULTS AND DISCUSSION

**Figure 1:**
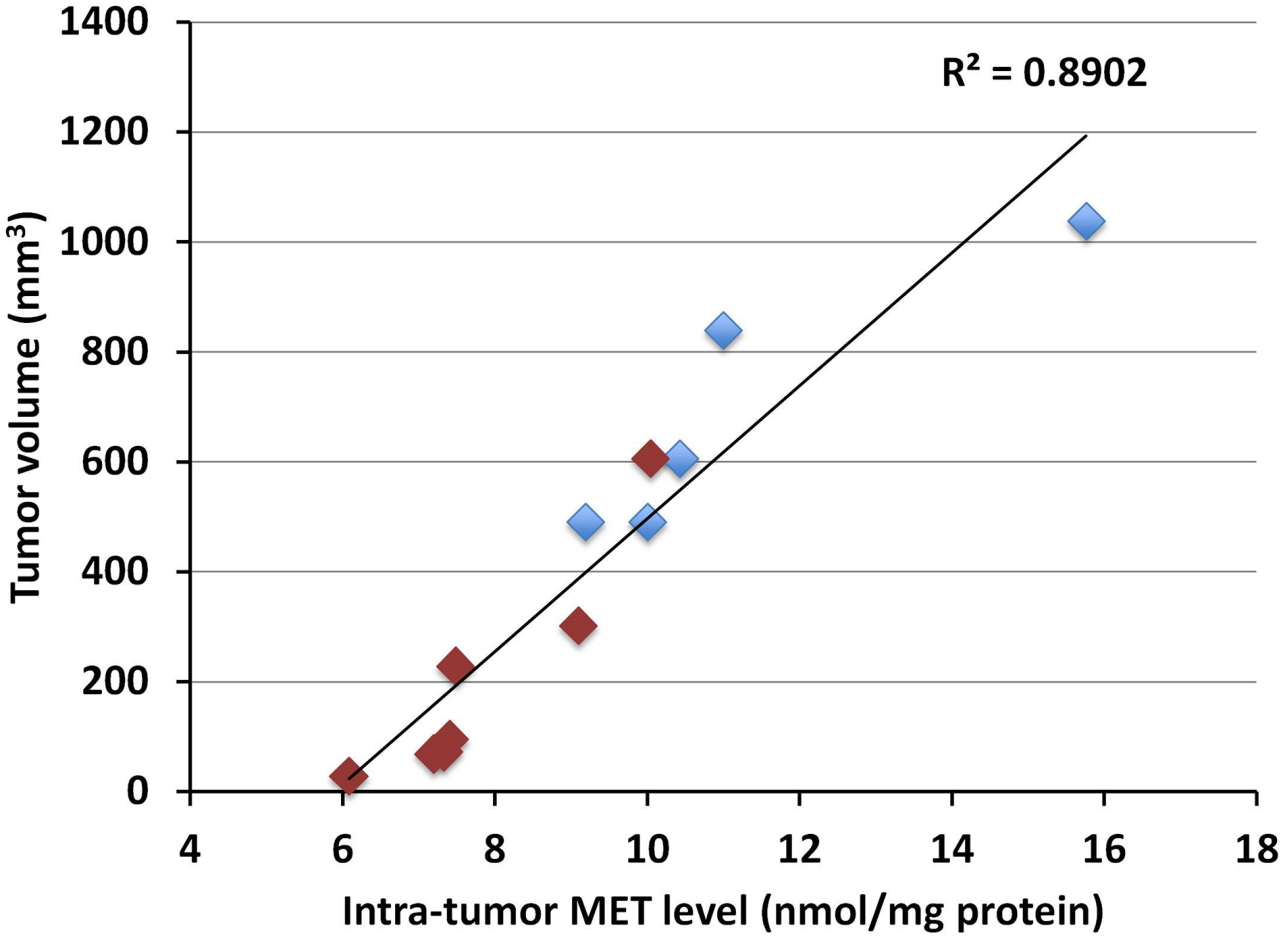
Correlation between tumor volume and intra-tumor MET level in the pancreatic cancer PDOX Blue box: untreated controls; Red box: treated with rMETase. Please see the Materials and Methods for details.

**Figure 2:**
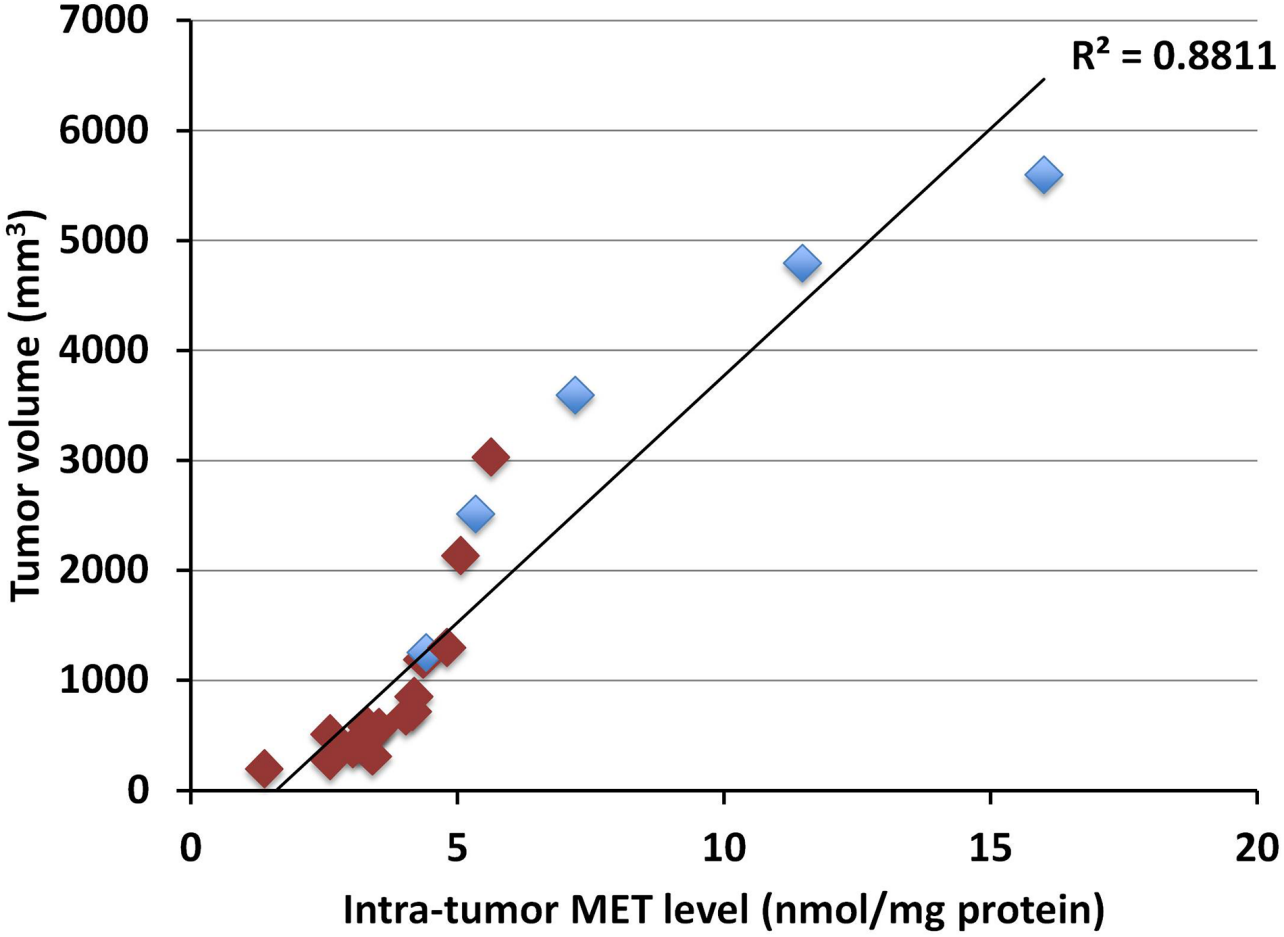
Correlation between tumor volume and intra-tumor MET level in the melanoma PDOX Blue box: untreated controls; Red box: treated with rMETase. Please see the Materials and Methods for details.

Intra-tumor MET levels highly correlated with tumor volume in both the pancreatic cancer (*p*<0.0001, R^2^=0.89016) (Figure 1) and melanoma PDOX models (*p*<0.0001, R^2^=0.88114) (Figure 2). Tumors with low concentration of MET were smaller in size. Tumors treated with rMETase had lower concentration of MET and were smaller in size than untreated tumors (Table 1).

**Table 1:** Intra-tumor MET levels (nmol/mg protein) and volume (mm^3^) after rMETase treatment

MET concentration			
	Untreated control	Treated with rMETase	*p*-value
Pancreatic cancer PDOX	11.3 ± 0.87	7.80 ± 0.73	*p* = 0.0006
Melanoma PDOX	8.88 ± 1.05	3.65 ± 0.57	*p* = 0.0003

Tumor volume			
	Untreated control	Treated with rMETase	*p*-value
Pancreatic cancer PDOX	693.9 ± 239.6	200.5 ± 204.2	*p* = 0.0032
Melanoma PDOX	3755.2 ± 484.3	857.9 ± 262.6	*p* < 0.0001

The present study shows a direct relationship between the intra-tumor MET level and tumor size using PDOX models of pancreatic cancer and melanoma, further demonstrating the MET dependence of cancer, in this case, using patient tumors.

The excessive requirement for MET termed MET dependence appears to be a general metabolic defect in cancer. Sugimura *et al.* showed that rat tumor growth was slowed by giving the rats a defined diet depleted in MET [27]. It was observed that L5178Y mouse leukemia cells in culture required very high levels of MET to proliferate [28]. Subsequently, most cancer cell lines were found to be MET dependent [29, 30]. These cell lines were derived from multiple cancer types including liver, ovarian, submaxillary, brain, lung, bladder, prostate, breast, kidney, cervical, colon, fibrosarcoma, osteosarcoma, rhabdomyosarcoma, leiomyosarcoma, neuroblastoma, glioblastoma, pancreatic and melanoma. The occurrence of MET dependence among these diverse cancer types suggests that methionine dependence is a general phenomenon in cancer. The present results further substantiate this assumption.

Human patient tumors, including tumors of the colon, breast, ovary, prostate, and melanoma, were previously found to be MET dependent in Gelfoam^®^ histoculture [31]. Mouse models of human cell lines were previously shown to be inhibited by rMETase [32–34].

PDOX models of Ewing’s sarcoma [35] and melanoma [26] were also shown to be MET dependent and inhibited by rMETase.

This is the first report that intra-tumor MET levels highly correlated with tumor volume. These results demonstrate that MET restriction, using rMETase, has promising clinical potential.

Previously-developed concepts and strategies of highly-selective tumor targeting can take advantage of molecular targeting of tumors, including tissue-selective therapy which focuses on unique differences between normal and tumor tissues [36–41].

## MATERIALS AND METHODS

### Mice

Athymic *nu/nu* nude mice (AntiCancer Inc., San Diego, CA), 4–6 weeks old, were used in this study. Mice were housed in a barrier facility on a high efficacy particulate arrestance (HEPA)-filtered rack under standard conditions of 12-hour light/dark cycles. The animals were fed an autoclaved laboratory rodent diet. All animal studies were conducted in accordance with the principles and procedures outlined in the National Institutes of Health Guide for the Care and Use of Animals under Assurance Number A3873-1. All mouse surgical procedures and imaging were performed with the animals anesthetized by subcutaneous injection of a ketamine mixture (0.02 ml solution of 20 mg/kg ketamine, 15.2 mg/kg xylazine, and 0.48 mg/kg acepromazine maleate). The response of animals during surgery was monitored to ensure adequate depth of anesthesia. The animals were observed on a daily basis and humanely sacrificed by CO_2_ inhalation if they met the following humane-endpoint criteria: severe tumor burden (more than 20 mm in diameter), prostration, significant body weight loss, difficulty breathing, rotational motion and body temperature drop [26].

### Patient-derived pancreatic cancer

The pancreatic tumor was established in nude mice at the MD Anderson Cancer Center under IRB approval and written informed patient consent [42–49].

### Surgical orthotopic implantation (SOI) of pancreatic cancer

For the pancreatic cancer PDOX, tumor fragments (5 mm^3^) were initially implanted subcutaneously in nude mice. After five weeks, the subcutaneously-implanted tumors grew to more than 10 mm in diameter. The subcutaneously-grown tumors were then harvested and cut into small fragments (3 mm^3^). After nude mice were anesthetized with the ketamine solution described above, a 1-2 cm skin incision was made on the left side abdomen through the skin, fascia and peritoneum and pancreas was exposed. Surgical sutures (8-0 nylon) were used to implant tumor fragments onto the tail of pancreas to establish the PDOX model. The wound was closed with a 6-0 nylon suture (Ethilon, Ethicon, Inc., NJ, USA) [50, 51].

### Patient-derived melanoma

The melanoma patient PDOX was previously established from a patient diagnosed with a melanoma of the right chest wall under UCLA IRB approval and written informed patient consent [26, 52–55].

### SOI of melanoma

After subcutaneously-implanted tumors grew to more than 10 mm in diameter, the subcutaneously-grown tumors were then harvested and cut into small fragments (3 mm^3^). After nude mice were anesthetized with the ketamine solution described above, a 5-mm skin incision was made on the right chest into the chest wall in order to match the patient, which was split to make space for the melanoma tissue fragment. A single tumor fragment was implanted orthotopically into the space to establish the PDOX model. The wound was closed with a 6-0 nylon suture (Ethilon, Ethicon, Inc., NJ, USA) [26, 52–55].

### Recombinant methionase (rMETase) production

Recombinant L-methionine α-deamino-γ-mercaptomethane lyase (recombinant methioninase [rMETase]) [EC 4.4.1.11] from *Pseudomonas putida* has been previously cloned and was produced in *Escherichia coli* (AntiCancer, Inc., San Diego, CA) [20, 23]. rMETase is a homotetrameric PLP enzyme of 172-kDa molecular mass [20, 25].

### Treatment study design

PDOX mouse models were randomized into two groups: untreated control; rMETase (100 units, i.p., 14 consecutive days). Tumor length and width were measured at post-treatment. Tumor volume was calculated with the following formula: Tumor volume (mm^3^) = length (mm) × width (mm) × width (mm) × ½ [26].

### Intra-tumor MET level analysis

Each tumor was sonicated for 30 seconds on ice and centrifuged at 12,000 rpm for 10 minutes. Supernatants were collected and protein levels were measured using the Coomassie Protein Assay Kit (Thermo Scientific, Rockford, IL). Protein levels were calculated from a standard curve obtained with a protein standard, bovine serum albumin (BSA). MET levels were determined with the HPLC procedure described previously. MET levels were calculated per mg tumor protein [26].

### Statistical analysis

JMP version 11.0 was used for analysis of variance (ANOVA). A probability value of *P* ≤ 0.05 was considered statistically significant.

## CONCLUSIONS

Currently melanoma [56–59] and pancreatic cancer [60, 61] are recalcitrant diseases with no reliable therapy. The results of the present study indicate that rMETase has general clinical potential to improve the outcome for both diseases as non-BRAF-V600E melanoma is also sensitive to rMETase [62].

## References

[1] Hoffman RM (2015). Development of recombinant methioninase to target the general cancer-specific metabolic defect of methionine dependence: a 40-year odyssey. Expert Opin Biol Ther.

[2] Guo H, Lishko VK, Herrera H, Groce A, Kubota T, Hoffman RM (1993). Therapeutic tumor-specific cell cycle block induced by methionine starvation *in vivo*. Cancer Res.

[3] Hoffman RM, Jacobsen SJ (1980). Reversible growth arrest in simian virus 40-transformed human fibroblasts. Proc Natl Acad Sci U S A.

[4] Kokkinakis DM, von Wronski MA, Vuong TH, Brent TP, Schold SC (1997). Regulation of O6-methylguanine-DNA methyltransferase by methionine in human tumour cells. Br J Cancer.

[5] Kokkinakis DM, Schold SC, Hori H, Nobori T (1997). Effect of long-term depletion of plasma methionine on the growth and survival of human brain tumor xenografts in athymic mice. Nutr Cancer.

[6] Hoffman RM, Erbe RW (1976). High *in vivo* rates of methionine biosynthesis in transformed human and malignant rat cells auxotrophic for methionine. Proc Natl Acad Sci U S A.

[7] Stern PH, Mecham JO, Wallace CD, Hoffman RM (1983). Reduced free-methionine in methionine-dependent SV40-transformed human fibroblasts synthesizing apparently normal amounts of methionine. J Cell Physiol.

[8] Stern PH, Wallace CD, Hoffman RM (1984). Altered methionine metabolism occurs in all members of a set of diverse human tumor cell lines. J Cell Physiol.

[9] Hoffman RM (1984). Altered methionine metabolism, DNA methylation and oncogene expression in carcinogenesis. A review and synthesis. Biochim Biophys Acta.

[10] Coalson DW, Mecham JO, Stern PH, Hoffman RM (1982). Reduced availability of endogenously synthesized methionine for S-adenosylmethionine formation in methionine-dependent cancer cells. Proc Natl Acad Sci U S A.

[11] Stern PH, Hoffman RM (1984). Elevated overall rates of transmethylation in cell lines from diverse human tumors. *In Vitro*.

[12] Warburg O (1956). On the origin of cancer cells. Science.

[13] Hoffman RM (2017). Is DNA methylation the new guardian of the genome?. Mol Cytogenet.

[14] Hoffman RM (2017). The wayward methyl group and the cascade to cancer. Cell Cycle.

[15] Xu W, Gao L, Shao A, Zheng J, Zhang J (2017). The performance of 11C-Methionine PET in the differential diagnosis of glioma recurrence. Oncotarget.

[16] Lishko VK, Lishko OV, Hoffman RM (1993). The preparation of endotoxin-free L-methionine-alpha-deamino-gamma-mercaptomethane-lyase (L-methioninase) from Pseudomonas putida. Protein Expr Purif.

[17] Lishko VK, Lishko OV, Hoffman RM (1993). Depletion of serum methionine by methioninase in mice. Anticancer Res.

[18] Tan Y, Zavala J, Xu M, Zavala J, Hoffman RM (1996). Serum methionine depletion without side effects by methioninase in metastatic breast cancer patients. Anticancer Res.

[19] Tan Y, Zavala J, Han Q, Xu M, Sun X, Tan X, Tan X, Magana R, Geller J, Hoffman RM (1997). Recombinant methioninase infusion reduces the biochemical endpoint of serum methionine with minimal toxicity in high-stage cancer patients. Anticancer Res.

[20] Tan Y, Xu M, Tan X, Tan X, Wang X, Saikawa Y, Nagahama T, Sun X, Lenz M, Hoffman RM (1997). Overexpression and large-scale production of recombinant L-methionine-alpha-deamino-gamma-mercaptomethane-lyase for novel anticancer therapy. Protein Expr Purif.

[21] Inoue H, Inagaki K, Sugimoto M, Esaki N, Soda K, Tanaka H (1995). Structural analysis of the L-methionine gamma-lyase gene from Pseudomonas putida. J Biochem.

[22] Hori H, Takabayashi K, Orvis L, Carson DA, Nobori T (1996). Gene cloning and characterization of Pseudomonas putida L-methionine-alpha-deamino-gamma-mercaptomethane-lyase. Cancer Res.

[23] Takakura T, Ito T, Yagi S, Notsu Y, Itakura T, Nakamura T, Inagaki K, Esaki N, Hoffman RM, Takimoto A (2006). High-level expression and bulk crystallization of recombinant L-methionine γ-lyase, an anticancer agent. Appl Microbiol Biotechnol.

[24] Takakura T, Takimoto A, Notsu Y, Yoshida H, Ito T, Nagatome H, Ohno M, Kobayashi Y, Yoshioka T, Inagaki K, Yagi S, Hoffman RM, Esaki N (2006). Physicochemical and pharmacokinetic characterization of highly potent recombinant L-methionine γ-lyase conjugated with polyethylene glycol as an antitumor agent. Cancer Res.

[25] Kudou D, Misaki S, Yamashita M, Tamura T, Takakura T, Yoshioka T, Yagi S, Hoffman RM, Takimoto A, Esaki N, Inagaki K (2007). Structure of the antitumour enzyme L-methionine γ-lyase from Pseudomonas putida at 1.8Å resolution. J Biochem.

[26] Kawaguchi K, Igarashi K, Li S, Han Q, Tan Y, Kiyuna T, Miyake Y, Murakami T, Chmielowski B, Nelson SD, Russell TA, Dry SM, Li Y (2017). Combination treatment with recombinant methioninase enables temozolomide to arrest a BRAF V600E melanoma growth in a patient-derived orthotopic xenograft. Oncotarget.

[27] Sugimura T, Birnbaum SM, Winitz M, Greenstein JP (1959). Quantitative nutritional studies with water-soluble, chemically defined diets. VIII. The forced feeding of diets each lacking in one essential amino acid. Arch Biochem Biophys.

[28] Chello PL, Bertino JR (1973). Dependence of 5-methyltetrahydrofolate utilization by L5178Y murine leukemia cells *in vitro* on the presence of hydroxycobalamin and transcobalamin II. Cancer Res.

[29] Mecham JO, Rowitch D, Wallace CD, Stern PH, Hoffman RM (1983). The metabolic defect of methionine dependence occurs frequently in human tumor cell lines. Biochem Biophys Res Commun.

[30] Tan Y, Xu M, Hoffman RM (2010). Broad selective efficacy of recombinant methioninase and polyethylene glycol-modified recombinant methioninase on cancer cells *in vitro*. Anticancer Res.

[31] Guo HY, Herrera H, Groce A, Hoffman RM (1993). Expression of the biochemical defect of methionine dependence in fresh patient tumors in primary histoculture. Cancer Res.

[32] Yoshioka T, Wada T, Uchida N, Maki H, Yoshida H, Ide N, Kasai H, Hojo K, Shono K, Maekawa R, Yagi S, Hoffman RM, Sugita K (1998). Anticancer efficacy *in vivo* and *in vitro*, synergy with 5-fluorouracil, and safety of recombinant methioninase. Cancer Res.

[33] Tan Y, Sun X, Xu M, Tan X, Sasson A, Rashidi B, Han Q, Tan X, Wang X, An Z, Sun FX, Hoffman RM (1999). Efficacy of recombinant methioninase in combination with cisplatin on human colon tumors in nude mice. Clin Cancer Res.

[34] Kokkinakis DM, Hoffman RM, Frenkel EP, Wick JB, Han Q, Xu M, Tan Y, Schold SC (2001). Synergy between methionine stress and chemotherapy in the treatment of brain tumor xenografts in athymic mice. Cancer Res.

[35] Murakami T, Li S, Han Q, Tan Y, Kiyuna T, Igarashi K, Kawaguchi K, Hwang HK, Miyaki K, Singh AS, Hiroshima Y, Lwin TM, DeLong JC (2017). Recombinant methioninase effectively targets a Ewing’s sarcoma in a patient-derived orthotopic xenograft (PDOX) nude-mouse model. Oncotarget.

[36] Blagosklonny MV (2003). Matching targets for selective cancer therapy. Drug Discov Today.

[37] Blagosklonny MV (2005). Teratogens as anti-cancer drugs. Cell Cycle.

[38] Blagosklonny MV (2001). Treatment with inhibitors of caspases, that are substrates of drug transporters, selectively permits chemotherapy-induced apoptosis in multidrug-resistant cells but protects normal cells. Leukemia.

[39] Blagosklonny MV (2006). Target for cancer therapy: proliferating cells or stem cells. Leukemia.

[40] Apontes P, Leontieva OV, Demidenko ZN, Li F, Blagosklonny MV (2011). Exploring long-term protection of normal human fibroblasts and epithelial cells from chemotherapy in cell culture. Oncotarget.

[41] Blagosklonny MV (2003). Tissue-selective therapy of cancer. Br J Cancer.

[42] Suetsugu A, Katz M, Fleming J, Moriwaki H, Bouvet M, Saji S, Hoffman RM (2012). Multi-color palette of fluorescent proteins for imaging the tumor microenvironment of orthotopic tumorgraft mouse models of clinical pancreatic cancer specimens. J Cell Biochem.

[43] Suetsugu A, Katz M, Fleming J, Truty M, Thomas R, Saji S, Moriwaki H, Bouvet M, Hoffman RM (2012). Imageable fluorescent metastasis resulting in transgenic GFP mice orthotopically implanted with human-patient primary pancreatic cancer specimens. Anticancer Res.

[44] Suetsugu A, Katz M, Fleming J, Truty M, Thomas R, Saji S, Moriwaki H, Bouvet M, Hoffman RM (2012). Non-invasive fluorescent-protein imaging of orthotopic pancreatic-cancer-patient tumorgraft progression in nude mice. Anticancer Res.

[45] Hiroshima Y, Zhao M, Maawy A, Zhang Y, Katz MH, Fleming JB, Uehara F, Miwa S, Yano S, Momiyama M, Suetsugu A, Chishima T, Tanaka K (2014). Efficacy of Salmonella typhimurium A1-R versus chemotherapy on a pancreatic cancer patient-derived orthotopic xenograft (PDOX). J Cell Biochem.

[46] Hiroshima Y, Maawy A, Zhang Y, Murakami T, Momiyama M, Mori R, Matsuyama R, Katz MH, Fleming JB, Chishima T, Tanaka K, Ichikawa Y, Endo I (2014). Metastatic recurrence in a pancreatic cancer patient derived orthotopic xenograft (PDOX) nude mouse model is inhibited by neoadjuvant chemotherapy in combination with fluorescence-guided surgery with an anti-CA 19-9-conjugated fluorophore. PLoS One.

[47] Hiroshima Y, Maawy AA, Katz MH, Fleming JB, Bouvet M, Endo I, Hoffman RM (2015). Selective efficacy of zoledronic acid on metastasis in a patient-derived orthotopic xenograph (PDOX) nude-mouse model of human pancreatic cancer. J Surg Oncol.

[48] Yano S, Hiroshima Y, Maawy A, Kishimoto H, Suetsugu A, Miwa S, Toneri M, Yamamoto M, Katz MH, Fleming JB, Urata Y, Tazawa H, Kagawa S (2015). Color-coding cancer and stromal cells with genetic reporters in a patient-derived orthotopic xenograft (PDOX) model of pancreatic cancer enhances fluorescence-guided surgery. Cancer Gene Ther.

[49] Hoover M, Adamian Y, Brown M, Maawy A, Chang A, Lee J, Gharibi A, Katz MH, Fleming J, Hoffman RM, Bouvet M, Doebler R, Kelber JA (2017). A novel method for RNA extraction from FFPE samples reveals significant differences in biomarker expression between orthotopic and subcutaneous pancreatic cancer patient-derived xenografts. Oncotarget.

[50] Fu X, Guadagni F, Hoffman RM (1992). A metastatic nude-mouse model of human pancreatic cancer constructed orthotopically with histologically intact patient specimens. Proc Natl Acad Sci U S A.

[51] Hoffman RM (1999). Orthotopic metastatic mouse models for anticancer drug discovery and evaluation: a bridge to the clinic. Invest New Drugs.

[52] Kawaguchi K, Murakami T, Chmielowski B, Igarashi K, Kiyuna T, Unno M, Nelson SD, Russell TA, Dry SM, Li Y, Eilber FC, Hoffman RM (2016). Vemurafenib-resistant BRAF-V600E mutated melanoma is regressed by MEK targeting drug trametinib, but not cobimetinib in a patient-derived orthotopic xenograft (PDOX) mouse model. Oncotarget.

[53] Kawaguchi K, Igarashi K, Murakami T, Chmiewloski B, Kiyuna T, Zhao M, Zhang Y, Singh A, Unno M, Nelson SD, Russell TA, Dry SM, Li Y (2016). Tumor-targeting Salmonella typhimurium A1-R combined with Temozolomide regresses malignant melanoma with a BRAF-V600 mutation in a patient-derived orthotopic xenograft (PDOX) model. Oncotarget.

[54] Kawaguchi K, Igarashi K, Murakami T, Kiyuna T, Zhao M, Zhang Y, Nelson SD, Russell TA, Dry SM, Singh AS, Chmielowski B, Li Y, Unno M (2017). Salmonella typhimurium A1-R targeting of a chemotherapy resistant BRAF-V600E melanoma in a patient-derived orthotopic xenograft (PDOX) model is enhanced in combination with either vemurafenib or temozlomide. Cell Cycle.

[55] Kawaguchi K, Igarashi K, Murakami T, Zhao M, Zhang Y, Chmielowski B, Kiyuna T, Nelson SD, Russell TA, Dry SM, Li Y, Unno M, Eilber FC (2017). Tumor-targeting Salmonella typhimurium A1-R sensitizes melanoma with a BRAF-V600E mutation to vemurafenib in a patient-derived orthotopic xenograft (PDOX) nude mouse model. J Cell Biochem.

[56] Slominski AT, Carlson JA (2014). Melanoma resistance: a bright future for academicians and a challenge for patient advocates. Mayo Clin Proc.

[57] Brożyna AA, Jóźwicki W, Roszkowski K, Filipiak J, Slominski AT (2016). Melanin content in melanoma metastases affects the outcome of radiotherapy. Oncotarget.

[58] Slominski AT, Brożyna AA, Zmijewski MA, Jóźwicki W, Jetten AM, Mason RS, Tuckey RC, Elmets CA (2017). Vitamin D signaling and melanoma: role of vitamin D and its receptors in melanoma progression and management. Lab Invest.

[59] Hoffman RM (2017). Patient-derived orthotopic xenograft (PDOX) models of melanoma. Int J Mol Sci.

[60] Von Hoff DD, Ervin T, Arena FP, Chiorean EG, Infante J, Moore M, Seay T, Tjulandin SA, Ma WW, Saleh MN, Harris M, Reni M, Dowden S (2013). Increased survival in pancreatic cancer with nab-paclitaxel plus gemcitabine. N Engl J Med.

[61] Matsuo Y, Raimondo M, Woodward TA, Wallace MB, Gill KR, Tong Z, Burdick MD, Yang Z, Strieter RM, Hoffman RM, Guha S (2009). CXC-chemokine/CXCR2 biological axis promotes angiogenesis *in vitro* and *in vivo* in pancreatic cancer. Int J Cancer.

[62] Kawaguchi K, Igarashi K, Li S, Han Q, Tan Y, Miyake K, Kiyuna T, Miyake M, Murakami T, Chmielowski S, Nelson SD, Russell TA, Dry SM (2018). Recombinant methioninase (rMETase) is an effective therapeutic for BRAF-V600E-negative as well as -positive melanoma in patient-derived orthotopic xenograft (PDOX) mouse models. Oncotarget.

